# Comparative Evaluation of Anticancer Activity of Natural Methoxylated Flavones Xanthomicrol and Eupatilin in A375 Skin Melanoma Cells

**DOI:** 10.3390/life14030304

**Published:** 2024-02-26

**Authors:** Antonella Rosa, Franca Piras, Federica Pollastro, Valeria Sogos, Giovanni Appendino, Mariella Nieddu

**Affiliations:** 1 Department of Biomedical Sciences, University of Cagliari, 09042 Monserrato, Italy; fpiras@unica.it (F.P.); sogos@unica.it (V.S.); mnieddu@unica.it (M.N.); 2 Department of Pharmaceutical Sciences, University of Eastern Piedmont “Amedeo Avogadro”, 28100 Novara, Italy; federica.pollastro@uniupo.it (F.P.); giovanni.appendino@uniupo.it (G.A.); 3 PlantaChem S.r.l.s., Via Amico Canobio 4/6, 28100 Novara, Italy

**Keywords:** methoxylated flavones, xanthomicrol, eupatilin, cancer cells, lipid profile modulation, apoptosis

## Abstract

Melanoma is a skin cancer caused by the malignant transformation of melanocytes and cutaneous melanoma represents the most aggressive and deadliest type of skin cancer with an increasing incidence worldwide. The main purpose of the present research was to evaluate the anticancer effects of the natural bioactive compounds xanthomicrol (XAN) and eupatilin (EUP) in human A375 malignant skin melanoma cells, a cell line widely used as an in vitro model of cutaneous melanoma. XAN and EUP are lipophilic methoxylated flavones with antioxidant, anti-inflammatory, and antitumor properties. The effects of XAN and EUP on cell viability, morphology, lipid profile, oxidative status, apoptosis, and mitochondrial membrane polarization were determined and compared in A375 cells. At 24 h-incubation (MTT assay), XAN significantly reduced viability at the dose range of 2.5–200 μM, while EUP showed a significant cytotoxicity from 25 μM. Moreover, both methoxylated flavones induced (at 10 and 25 μM, 24 h-incubation) marked cell morphological alterations (presence of rounded and multi-nucleated cells), signs of apoptosis (NucView 488 assay), and a noteworthy mitochondrial membrane depolarization (MitoView 633 assay), coupled to a marked lipid profile modulation, including variations in the ratio of phospholipid/cholesterol and a decrease in the oleic, palmitic, and palmitoleic acid amounts. Moreover, a remarkable time-dependent ROS generation (2′,7′-dichlorodihydrofluorescein diacetate assay) was observed during 3 h-incubation of A375 cancer cells in the presence of XAN and EUP (10 and 25 μM). Our results confirm the potential antitumor effect of natural EUP and XAN in cutaneous melanoma by the activation of multiple anticancer mechanisms.

## 1. Introduction

There has been a constant attempt to identify bioactive compounds from natural sources and establish their therapeutic effects and potential use in health promotion [1,2]. The modern drug discovery process is greatly based on the knowledge derived from herbal traditional medicine [3]. Phenolic compounds from plants, including phenolic acids, coumarins, flavonoids, stilbenes, and lignans, have attracted continuously increasing attention for their various biological activities [4,5,6]. Among natural polyphenolic compounds, flavonoids (flavones, flavanones, flavonols, flavan-3-ols, isoflavones, and anthocyanidins) are structurally characterized by C6-C3-C6 rings with different substitution patterns and exhibit antioxidant, anticarcinogenic, cardioprotective, antiallergenic, anti-inflammatory, and antimutagenic activity [4,6]. Flavonoids found in the highest amounts in the human diet include isoflavones, flavonols, and flavones [7].

**Figure 1:**
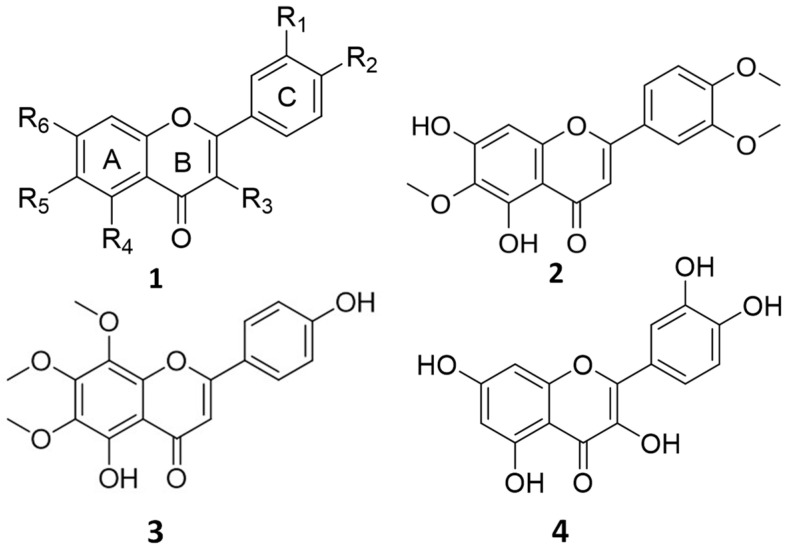
Chemical structure of flavones (**1**), eupatilin (EUP, **2**), xanthomicrol (XAN, **3**), and quercetin (QRC, **4**).

Flavones (Figure 1) are phenolic compounds that possess a 2-phenylchromen-4-one backbone with various types of substituents (hydroxyl, prenyl, methoxyl, and glycosyl groups) [6,7]. Numerous biological activities of flavones have been reported in the literature, such as anti-inflammatory, neuroprotective, antimicrobial, and anticancer activities [4,6,7,8]. The position and number of hydroxyl -OH and methoxyl -OCH_3_ groups on the flavone backbone greatly affect its bioactivity [9]. Various flavones exhibit potential therapeutic benefits in treating cancer [4,8,9,10,11,12]. Methoxylated flavones, uncommon plant lipophilic phenolic compounds, have been demonstrated to possess chemopreventive properties superior to unmethylated flavonoids or polyphenols [9]. Derivatives obtained from methylation of free phenolic groups (-OH) on the flavone skeleton are not susceptible to conjugation with sulfate or glucuronic acid, improving the ability to cross cell membranes, oral bioavailability, and metabolic stability [10,11].

The O-methylated flavones eupatilin (5,7-dihydroxy-3′,4′,6-trimethoxyflavone) (EUP, 2) and xanthomicrol (5,4′-dihydroxy-6,7,8-trimethoxyflavone) (XAN, 3) are naturally occurring pharmacologically active compounds (Figure 1) [13,14].

EUP is a lipophilic compound (the partition coefficient for the octanol–water mixture: log P3 = 2.9, Table 1) [15] with two free hydroxyl groups in the A ring (Figure 1).

EUP represents the major lipophilic flavonoid of plants belonging to the *Artemisia* species [16,17,18,19]. This flavone is especially found, in concentrations ranging from 100 to 200 mg/kg of dry weight, in the commonly known génépi plant groups including *Artemisia umbelliformis* Lam., *Artemisia genipi* Weber, and *A. eriantha*, the mountain wormwoods used for the production of the popular alpine liqueur genepy [13,16,17]. It was also identified in plants of *Centaurea*, *Tanacetum*, *Stachys*, and *Salvia* species [18]. EUP possesses a wide range of biological activities, including anti-inflammatory [18,19], antioxidant [13,18,20,21], and antitumor properties [13,18,22,23,24,25]. This phenol has been indicated as a potential therapeutic/chemopreventive agent for the treatment of different types of cancer [18]. The main mechanisms of EUP antitumor activity include the inhibition of cancer cell growth and proliferation [13,18,22,23,24,25], induction of apoptosis [13,18,24] cell differentiation [25], cell cycle arrest [14,22,23,25], mitochondria membrane potential reduction [13,18], alteration of cytoskeletal organization [26], and modulation of cancer cell signaling pathways [18,22,23,24]. Moreover, its anticancer activity has been reported to be mediated through its prooxidant activity [18].

The lipophilic compound XAN is characterized by two -OH groups as substituents on the A and C aromatic ring and a log P3 = 2.9 (Table 1) [15]. This methoxylated flavone is a bioactive compound present in several plants and herbs with a long application in traditional medicine, such as *Varthemia iphionoides*, *Achillea erba-rotta* subsp. *moschata* (Wulfen) I. Richardson, *Clinopodium douglasii*, *Dracocephalum kotschyi* Boiss, *Baccharis pentlandii* D.C., *Baccharis densiflora* Wedd, *Ocimum gratissimum* L., and *Artemisia campestris* L. [9,14,27,28,29,30]. XAN has antioxidant [9], antimicrobial [9], anti-inflammatory [9], and anticancer properties [9,14,27,28,29,30]. The XAN antitumor properties are linked to its capacity to inhibit the proliferation and viability in cancer cells [9,14,27,28,29], angiogenesis [27], cancer-related enzymes [27,30], and to induce apoptosis [9,14,27,30] and cell cycle arrest [9,14,27,30].

Cancer cells are characterized by a deregulation of lipid metabolism [31,32] and targeting lipid metabolism is considered an important therapeutic anticancer strategy [32,33,34]. Changes in the composition of lipid compounds in cancer cells affect the membrane fluidity, lipid rafts organization, and protein dynamics, leading to the induction of apoptotic pathways [35]. The antitumoral properties of flavones have been related to their ability to inhibit the expression of enzymes involved in lipid metabolism and alter the fluidity, structure, and organization of lipid membranes [13,14,36,37]. We recently demonstrated the capacity of EUP and XAN to impact lipid metabolism in cancer HeLa cells, specifically evidencing the inhibitory effect of both methoxylated flavones on the tumor cell de novo lipogenesis and desaturation [13,14].

Starting from all these considerations, this manuscript aimed to investigate and compare for the first time the modulatory effect of EUP and XAN on the lipid profile in cutaneous melanoma cells. Melanoma is a skin cancer caused by the malignant transformation of melanocytes [38]. Cutaneous melanoma represents the most aggressive and deadliest type of skin cancer (accounting for 5% of total skin cancers), with an increasing incidence worldwide [39,40,41]. It is one of the most therapy-resistant types of cancer [38]; therefore, the development of new therapeutic approaches is strongly mandatory. Lipid metabolic dysregulations have been demonstrated to contribute to the phenotype plasticity of melanoma cells and/or melanoma aggressiveness [42]. The increased rate of lipid synthesis (sterols and complex lipids) is the most prominent metabolic reprogramming feature that contributes to the pathogenesis/heterogeneity of melanoma [42]. Various alterations in fatty acid metabolism are present in melanoma cells, in particular, the upregulation of the expression of fatty acid synthase (FAS) has been evidenced in human melanoma [42]. Moreover, various pharmacological compounds known to target enzymes or receptors linked to lipid metabolism exhibited potential anticancer roles in different melanoma models [42].

In this study, we explored the ability of EUP and XAN to affect cell lipid metabolism, as another possible anticancer mechanism, in human diploid immortalized skin malignant melanoma (A375) cells, a tumor cell line amply used to assess the anticancer properties of compounds/extracts from natural sources in cutaneous melanoma [39,43]. Changes in the cell profile of phospholipids (PL), free cholesterol (FC), and total fatty acids (TFA) were examined in A375 melanoma cells after 24 h of incubation with EUP and XAN. Contemporaneously, experiments were designed to investigate the effect of the two methoxy flavones on cancer cell growth, cell morphology, apoptosis, mitochondria membrane potential, and reactive oxygen species (ROS) generation. The effect of EUP and XAN on cell viability and intracellular ROS formation was also monitored in a human keratinocyte cell line (HaCaT cells) for comparison.

## 2. Materials and Methods

### 2.1. Chemicals and Reagents

Standards of cholesterol, fatty acids, phosphocholines (PC 20:5/20:5, 18:2/18:2, 16:0/20:4, 16:0/18:2, 16:0/16:0, 18:1/18:1, 16:0/18:1, 18:1/16:0), 3-(4,5-dimethylthiazol-2-yl)-2,5-diphenyltetrazolium bromide (MTT), quercetin (QRC, purity ≥ 95%), and all solvents (purity ≥ 99.9%) were purchased from Sigma-Aldrich (Milan, Italy). All cell culture reagents/materials were provided by Invitrogen (Life Technologies, Milan, Italy). 2′,7′-Dichlorodihydrofluorescein diacetate (H_2_-DCF-DA) was purchased from Merck Life Science (Milan, Italy), while NucView^®^ 488 and MitoView™ 633 Apoptosis Assay Kit were obtained from Biotium (Fremont, CA, USA). EUP (98% purity) and XAN (98% purity) were isolated from the Swiss chemotype of *A. umbelliformis* Lam. (Asteraceae) as previously reported [13,17] and *A. erba-rotta* subsp. *moschata* (Wulfen) I. Richardson (musk yarrow, flowering tops) [14], respectively, and characterized according to the literature.

### 2.2. Cell Cultures

Human malignant A375 melanoma cells were purchased from the American Type Culture Collection (ATCC, Rockville, MD, USA). HaCaT cell line, a spontaneously immortalized human keratinocyte cell line from adult skin, was obtained by CLS-Cell Line Services (Eppelheim, Germany). Both cell lines were grown in Dulbecco’s modified Eagle’s medium (DMEM) with high glucose, supplemented with fetal calf serum (FCS) (10% *v*/*v*) and 2 mM L-glutamine, penicillin (100 units/mL)–streptomycin (100 μg/mL), at 37 °C in a 5% CO_2_ incubator. Subcultures of A375 and HaCaT cells were grown in T-75 culture flasks and passaged with a trypsin-EDTA solution.

### 2.3. Evaluation of Cytotoxic Effect by MTT Viability Assay

The cytotoxic effect of EUP and XAN cytotoxic was measured in A375 melanoma and HaCaT cells by the MTT viability colorimetric assay [13,14,44]. Cells were seeded (at a density of 3 × 10^4^ cells/mL for A375 cells and 10^5^ cells/mL for HaCaT cells) in 96-well plates in a complete culture medium (100 μL). After 48 h-incubation, cells were treated (for 24 h) in a fresh medium with various concentrations (2.5–200 μM) of EUP and XAN from dimethyl sulfoxide (DMSO) solutions (treated cells). The effect on A375 and HaCaT cell viability of different amounts of DMSO (molecule vehicle used to dissolve the phenolic compounds) was also evaluated. Then control (non-treated) cells, EUP- and XAN-treated cells, and cells incubated for 24 h with DMSO (vehicle-treated cells) were subjected to an MTT viability test as previously reported [44]. The flavone quercetin (QRC) (Figure 1) was also tested for cytotoxicity in A375 melanoma cells, at the doses 2.5–200 μM, as a well-known reference anticancer compound [13]. The auto microplate reader (Infinite 200, Tecan, Austria) was used to measure color development at the wavelength of 570 nm. The absorbance measured in each well was proportional to the number of viable cells and results were expressed as a percentage of cell viability compared to control cells.

Morphological observations of control cancer A375 cells and cells after 24 h of incubation with various amounts (2.5–200 μM) of EUP, XAN, and the reference compound QRC were performed by microscopic analysis with a ZOE™ Fluorescent Cell Imager (Bio-Rad Laboratories, Inc., Hercules, CA, USA).

### 2.4. Determination of Cell ROS Generation

Then, the EUP and XAN effect on the mitochondrial redox status of A375 melanoma cells was determined. The fluorogenic biosensor 2′,7′-dichlorodihydrofluorescein diacetate (H_2_-DCF-DA) was used to monitor the EUP- and XAN-induced intracellular ROS production as previously reported [45,46]. The effect of the two flavones on intracellular ROS generation was also monitored in normal skin HaCaT cells for comparison. Briefly, A375 cancer cells and HaCaT keratinocytes were cultured for 48 h (at a density of 3 × 10^4^ cells/ml for A375 cells and 10^5^ cells/mL for HaCaT cells) in 96-well plates in 100 μL of a complete culture medium. Cells (at 80% confluence) were then incubated for 30 min with 10 μM H_2_-DCF-DA in phosphate-buffered saline (PBS) solution at 37 °C. Afterwards, cells were washed and incubated for 3 h in fresh PBS in the absence (control cells) and in the presence of EUP and XAN 10 and 25 μM (from DMSO solutions) (treated cells), or the presence of 0.25% of DMSO (vehicle-treated cells). The H_2_-DCF-DA reagent, after penetration through the cell membrane, is deacetylated by cytoplasmic esterases, releasing the reduced state of 2,7-dichlorodihydrofluorescein (H_2_-DCF). The oxidation of the nonfluorescent moiety H_2_-DCF by intracellular ROS produces the highly fluorescent compound DCF [45,46]. ROS production was detected and monitored (every 5 min) for 3 h by using an Infinite 200 Tecan microplate reader at a controlled temperature of 37 °C. The reading was performed using an excitation wavelength of 490 nm and an emission wavelength of 520 nm. The Tecan I-control 1.5 V software was used for data collection and analysis. Fluorescence data were normalized to the respective control cells.

### 2.5. Experiments of Cell Lipid Profile Modulation

A375 melanoma cells were seeded (in T-75 culture flasks at a density of 3 × 10^5^ cells) in 10 mL of complete culture medium and cultured for 48 h. Then melanoma cells were incubated for a further 24 h in the absence (control cells) or the presence of EUP and XAN (10 and 25 μM, from a 10 mM solution in DMSO) (treated cells) in a fresh culture medium. These two amounts of EUP and XAN were selected to determine their effects on lipid profile at a low level of cell mortality and cell function compromise. Cells incubated for 24 h with DMSO 0.25% (vehicle-treated cells) were also prepared. A375 cells from different treatments were then washed, scraped, and centrifuged (10 min at 2000 rpm and 4 °C), and cell pellets were used for the lipid compound extraction.

### 2.6. Cell Lipid Extraction and Analysis

Total lipids were extracted from A375 melanoma cell pellets with 6 mL of the MeOH/chloroform/water 1:2:1 mixture as previously described [44]. The direct analysis of phospholipids (PL) and free cholesterol (FC) in the chloroform fractions after cell pellet extraction was performed with an Agilent Technologies 1100 HPLC system equipped with a 1260 Infinity evaporative light scattering detector (ELSD) and a diode array detector (DAD), as previously reported [47]. Methanol as a mobile phase (flow rate of 0.7 mL/min) and an Inertsil ODS-2 column (Superchrom, Milan, Italy) were used for PL and FC analysis (ELSD detection) [47]. Standard mixtures containing FC, polyunsaturated (P-PL), and saturated/monounsaturated (S/M-PL) phosphatidylcholines (PC) were used to assign the chromatographic region of each lipid class. The separation of lipid compounds was performed based on ECN (=CN − 2*n*, where CN indicates the number of acyl group carbons and n is the number of double bonds) [47].

Another aliquot of chloroform fractions after cell pellet extraction was dried and saponified in mild conditions for FA preparation as previously described [47]. The Agilent Technologies HPLC-DAD/ELSD system and the mixture acetonitrile/water/acetic acid (75/25/0.12, *v*/*v*/*v*) as mobile phase (flow rate of 2.3 mL/min) were used for the analysis of unsaturated (DAD detection, 200 nm) and saturated (ELSD detection) FA as previously reported. The Agilent OpenLAB Chromatography data system was used for data collection/analysis and linear (DAD) and quadratic (ELSD) calibration curves (correlation coefficients > 0.995) were constructed for FA quantification using standard compounds according to the literature [47].

### 2.7. Apoptosis and Mitochondrial Activity Assay

Then, the effect of EUP and XAN on apoptosis and mitochondrial membrane potential in A375 cancer cells was evaluated through the NucView^®^ 488 and MitoView™ 633 Apoptosis Assay Kit, as previously reported [45]. The NucView 488 is a cell membrane-permeable fluorogenic caspase substrate that crosses the cellular membrane and when cleaved by caspase-3/7, releases in the cytoplasm a dye that migrates into the nucleus and stains DNA with green fluorescence [45]. MitoView 633 is a far-red fluorescent dye with the ability to accumulate within mitochondria in a membrane potential-dependent manner [45,48]. Cells were seeded (at a density of 3 × 10^4^ cells/mL) in 96-well plates in 100 μL of complete culture medium and cultured for 48 h. Then, cells were incubated for 24 h in a complete medium in the absence (control cells) and the presence of EUP and XAN (10 and 25 μM, from DMSO solutions) (treated cells). An equivalent volume of DMSO (0.25%) was added to vehicle-treated cells. After incubation, the medium was carefully removed and cells were treated with NucView 488 and MitoView 633 probes in fresh medium, according to the manufacturer’s instructions and then incubated at 37 °C. The microscopic observations were finally made after 4 h (MitoView™ 633) and 14 h (NucView^®^ 488) of incubation using a ZOE™ Fluorescent Cell Imager. Instrument gain and offset values were adjusted using control (untreated) cells and remained constant for all subsequent experiments. ImageJ software (version 1.53e) was used for image analysis. Background fluorescence was subtracted from images and fluorescence intensity was expressed as % of the control cell fluorescence. Per each sample, 6 images (from different experiments) were processed for image analysis.

### 2.8. Statistical Analyses

Results were expressed as mean ± standard deviation and Graph Pad INSTAT 3.3 software (GraphPad Software, San Diego, CA, USA) was used for the evaluation of statistical differences between various data groups (treatments). Multiple comparisons of the group means were assessed by one–way analysis of variance (One-way ANOVA) followed by the Bonferroni Multiple Comparisons Test. Student’s unpaired *t*-test with Welch’s correction, which does not require the assumption of equal variance between populations, was used to compare the means of two data groups. The minimal level of significance was *p* < 0.05.

## 3. Results

### 3.1. Cytotoxic Activity (MTT Assay)

**Figure 2:**
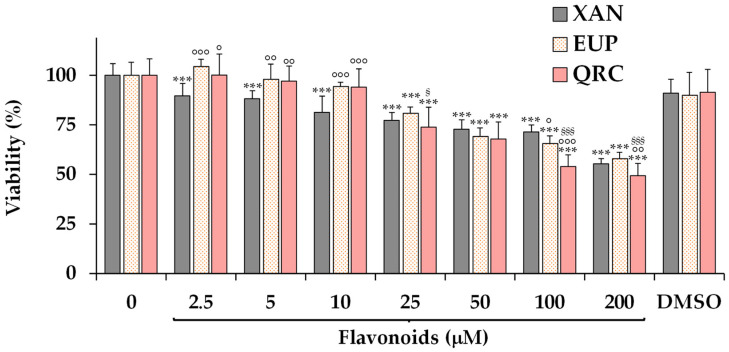
Values of viability, expressed as % of the control (0), induced by 24 h-incubation with different concentrations (2.5–200 μM) of xanthomicrol (XAN), eupatilin (EUP), the reference anticancer flavone quercetin (QRC), and the maximal non-toxic vehicle dose (DMSO 2%) in cancer A375 cells (MTT assay). All data are presented as mean (*n* = 12) and standard deviation. Statistical significance of differences was assessed by One-way ANOVA and Bonferroni post Test. For each series: *** = *p* < 0.001 versus control. For each concentration group: °°° = *p* < 0.001, °° = *p* < 0.01, ° = *p* < 0.05 versus XAN-treated cells; ^§§§^ = *p* < 0.001, ^§^ = *p* < 0.05 versus EUP-treated cells.

Firstly, the XAN and EUP cytotoxic activity was explored in A375 cells, a tumor cell line previously used to investigate the cytotoxicity of natural flavonoids [39,43]. The values of viability (expressed as % of the control) induced by 24 h-treatment with different concentrations (from 2.5 to 200 μM) of EUP and XAN in melanoma A375 cells by the MTT cell viability assay are reported in Figure 2.

The cytotoxic effect of the reference anticancer flavone QRC (2.5–200 μM) [13,49] is also reported for comparison in Figure 2.

XAN significantly (*p* < 0.001) affected A375 cell growth from the dose of 2.5 μM, with values of viability reduction, in comparison with control cells, increasing in a dose-dependent manner from 11% at 2.5 μM to 45% at the highest tested dose (200 μM). At the dose range of 25–100 μM, EUP showed a cancer cell growth inhibition of 20–34%, while a 42% reduction in viability was determined at the highest tested dose (200 μM). XAN was significantly more active than EUP in reducing A375 cell growth at low concentrations (2.5–10 μM), while similar toxicity values were observed from the dose of 25 μM. DMSO, the solvent used to dissolve the compounds, did not show a significant toxic effect, in comparison with control cells, in A375 cells in the range dose of 0.025–2%, showing a slight, statistically non-significant 8–9% viability reduction at the maximal tested dose (2%, Figure 2). In our experimental conditions, it was not possible to determine the exact IC_50_ value (the concentration of compound that induces a 50% cell viability decrease) because it exceeded the maximum percentage (2%) of DMSO tolerated in A375 melanoma cells. QRC, the reference anticancer flavone (log P3 = 1.5, as reported in Table 1) [13,15,49], was less toxic in cancer A375 cells than XAN in the range 2.5–10 μM; however, it showed values of cancer cell viability reduction similar to XAN and EUP at 25 and 50 μM. At the highest tested doses (100 and 200 μM), the well-known anticancer flavone QRC was slightly more active than the two methoxylated flavones, showing an IC_50_ value after 24 h-incubation of 184 μM.

**Figure 3:**
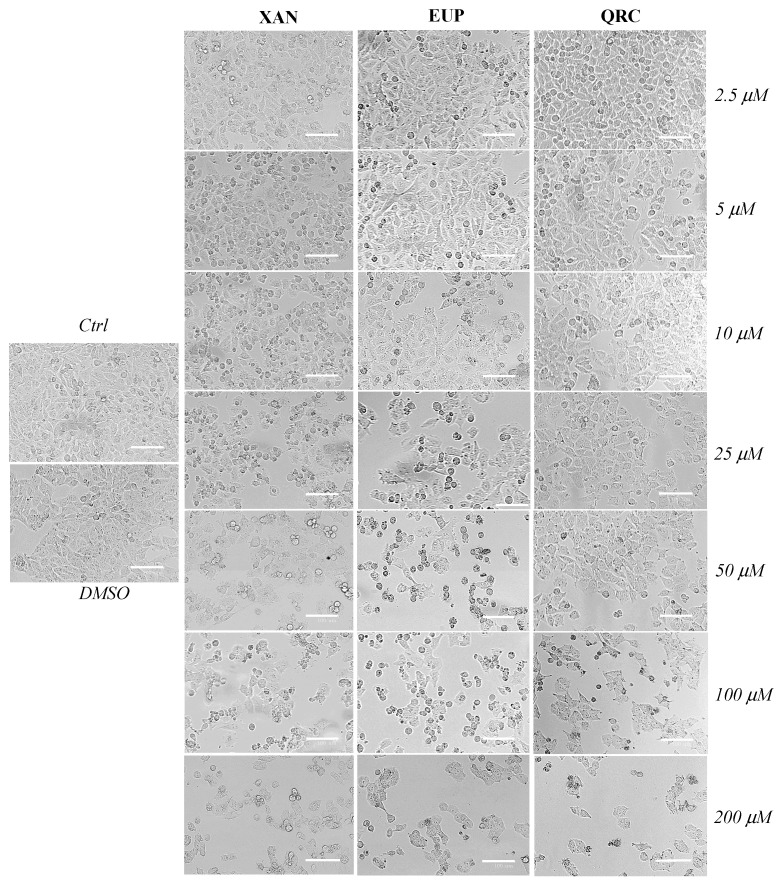
Phase contrast images of control A375 cancer cells, vehicle-treated cells (DMSO 2%, 24 h-incubation), and cells incubated for 24 h with different amounts (2.5–200 μM) of xanthomicrol (XAN), eupatilin (EUP), and quercetin (QRC). Bar = 100 μm.

Microscopic observation of A375 melanoma cells treated with XAN and EUP for 24 h, before the MTT assay, allowed us to evidence marked changes in cell morphologies compared to control cells (Figure 3).

In the control group, A375 melanoma cells were small, shuttle-shaped with evident borders, and closely linked to each other (tightly packed). Moreover, a high number of mitotic cells was observed. The treatment with XAN and EUP induced a remarkable concentration-dependent decrease in cell density and a rise in the number of rounded cells (apoptotic cells), evident from 5 μM and 10 μM for XAN and EUP, respectively. Moreover, the occurrence of clear apoptotic bodies, pyknotic nuclei, and cell debris was observed from 25 μM of both compounds. The microscopic observation of A375 cells treated with the reference compound QRC did not show an evident cell morphology and density alteration in the dose range of 2.5–10 μM, while changes in cell size and areas with a decreased cell density and cell-to-cell packing were noted from QRC 25 μM.

**Figure 4:**
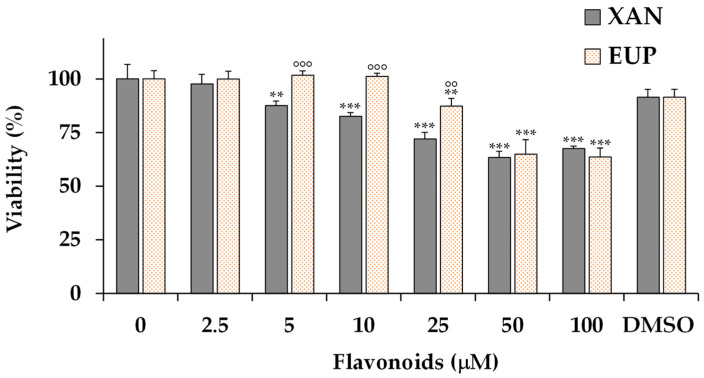
Values of viability, expressed as % of the control (0), induced by 24 h-incubation with different concentrations (2.5–100 μM) of xanthomicrol (XAN), eupatilin (EUP), and the maximal non-toxic vehicle dose (DMSO 1%) in normal HaCaT keratinocytes (MTT assay). All data are presented as mean (*n* = 9) and standard deviation. For each series: *** = *p* < 0.001, ** = *p* < 0.01 versus control (One-way ANOVA and Bonferroni post Test). For each concentration group: °°° = *p* < 0.001, °° = *p* < 0.01 versus XAN-treated cells (Student’s unpaired t-test with Welch’s correction).

Then, the cytotoxic effect of EUP and XAN was also investigated on human HaCaT keratinocytes, a normal skin cell line. Figure 4 shows the viability, expressed as % of the control, induced by the 24 h treatment with different amounts (2.5–100 μM) of XAN and EUP in HaCaT cells by MTT assay.

As observed in A375 melanoma cells, XAN was more toxic than EUP in HaCaT keratinocytes. No changes in cell viability, with respect to control cells, were observed in HaCaT keratinocytes treated with XAN at 2.5 μM, showing a certain selective toxicity towards malignant cells. A significant viability reduction, ranging from 12 to 32%, was observed for XAN in HaCaT cells from the dose of 5 μM to 100 μM.

EUP showed no cytotoxicity in the range of 2.5–10 μM and a 12% viability inhibition at 25 μM, evidencing at this dose a lower toxicity than in cancer A375 cells. A significant viability reduction of 35 and 36% was observed at 50 and 100 μM, respectively.

The vehicle DMSO did not show a significant toxic effect, versus control cells, in HacaT cells in the range dose of 0.025–1%, showing a non-significant, slight 8% viability reduction at the maximal tolerable tested dose (1%, Figure 4).

Table 2 shows the cytotoxic activity of EUP and XAN previously assessed in several normal and cancer cell lines [13,14,22,23,28,30].

The cytotoxic profile of XAN and EUP in A375 cancer cells was comparable to that previously reported for both compounds in human cervical cancer cells HeLa after 24 h of incubation, with XAN showing superior cytotoxicity than EUP at the lowest tested concentrations but a similar growth inhibitory effect from the dose of 50 μM [13,14].

The concentrations of 10 μM (81 and 94% of viability for XAN and EUP, respectively) and 25 μM (77–80% of viability, apoptotic morphology of most cells) were selected for both flavones for successive experiments in A375 melanoma cells.

### 3.2. Determination of Cell ROS Generation

**Figure 5:**
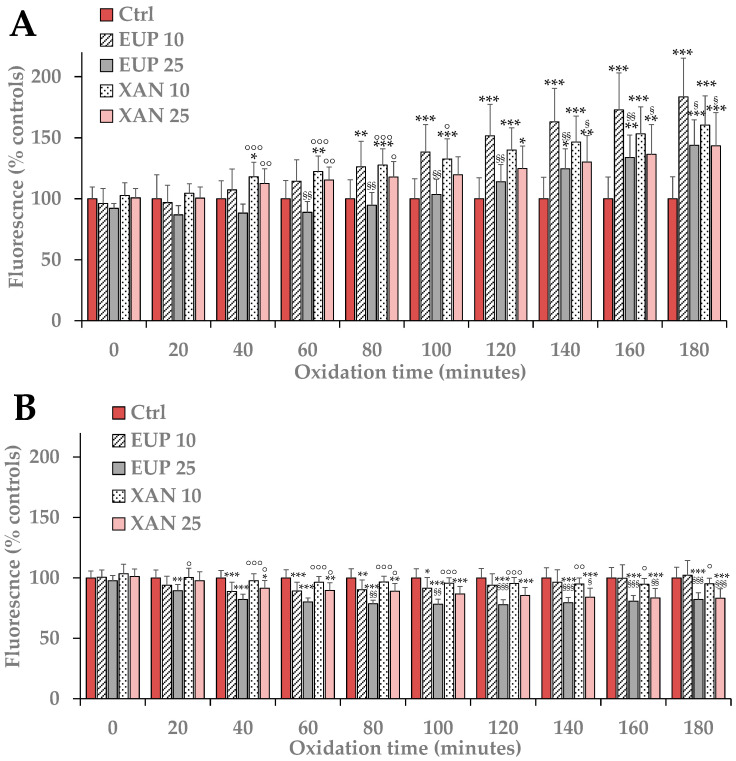
ROS-induced fluorescence, expressed as % of the control (0), measured at different time points in melanoma A375 cells (**A**) and normal HaCaT keratinocytes (**B**) during 3 h of incubation with eupatilin (EUP) and xanthomicrol (XAN) 10 and 25 μM. All data are presented as mean (*n* = 9) and standard deviation. At each time point: *** = *p* < 0.001, ** = *p* < 0.01, * = *p* < 0.05 versus control (0); ^§§§^ = *p* < 0.001, ^§§^ = *p* < 0.01, ^§^ = *p* < 0.05 versus cells treated with EUP 10 μM; °°° = *p* < 0.001, °° = *p* < 0.01, ° = *p* < 0.05 versus cells treated with EUP 25 μM. Statistical significance of differences was evaluated by One-way ANOVA and Bonferroni post Test.

Subsequently, differences in the mitochondrial redox status after the treatment with EUP and XAN were measured in A375 melanoma cells. The effect of the two flavones on ROS generation was also evaluated in normal skin HaCaT cells for comparison. Cancer A375 cells and HaCaT keratinocytes were incubated for 3 h with EUP and XAN 10 and 25 μM and the H_2_-DCF-DA assay [46] was used to monitor the time-dependent intracellular ROS generation during the treatment in comparison to control cells (Figure 5).

The treatment with EUP and XAN induced in A375 melanoma cells (Figure 5A), during 3 h of incubation, a time-dependent increase in the cell fluorescence compared to the basal rate of control cells. In general, at each time point, the ROS production was more evident at the lowest dose for both compounds. Moreover, the highest ROS generation was observed in cells treated with EUP 10 μM at 3 h-incubation. DMSO, the solvent employed to dissolve the compounds, did not induce, in comparison to control melanoma cells, ROS generation.

Interestingly, EUP and XAN did not exert a prooxidant effect on normal HaCaT keratinocytes during 3 h-incubation (Figure 5B). HaCaT cells incubated with both flavones showed the same basal ROS level as control cells at the dose of 10 μM, whereas a significant decrease in the cell fluorescence compared to control cells was observed at 25 μM, indicating a protective effect on keratinocytes.

Our data evidenced a selective ROS generation in A375 cells, qualifying prooxidant effects as a potential mechanism of EUP and XAN toxicity in cancer cells.

### 3.3. Modulatory Effect on Cell Lipids

Then, the modulatory effect of XAN and EUP 10 and 25 μM on polar lipid classes and the total fatty acids profile of cancer A375 cells were assessed after 24 h of incubation. Cell pellets obtained from control and treated A375 cells were subjected to the extraction of lipid components, and aliquots of lipid extracts were directly analyzed for the content of different lipid classes such as FC, saturated/monounsaturated phospholipids (S/M-PL), and polyunsaturated phospholipids (P-PL).

**Figure 6:**
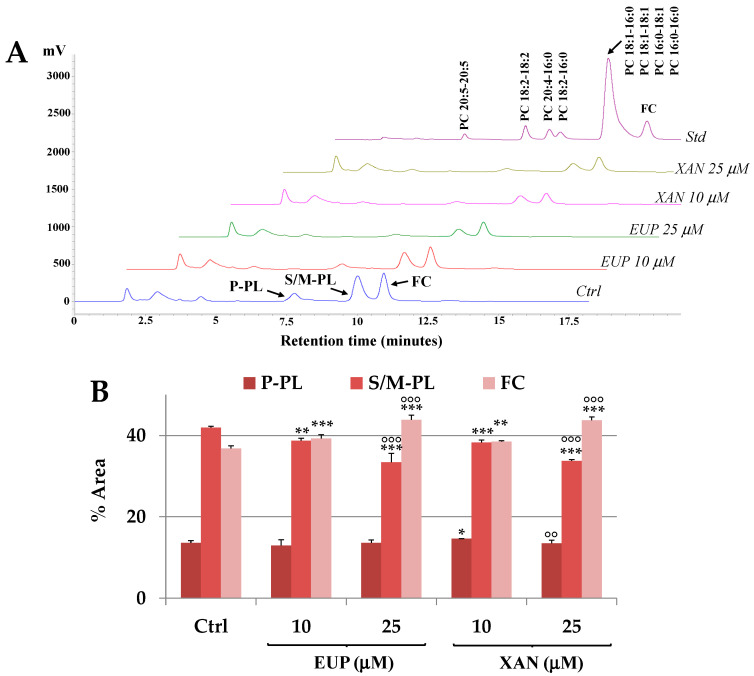
(**A**) Chromatographic profile obtained by HPLC-ELSD analysis of free cholesterol (FC), polyunsaturated phospholipids (P-PL), and saturated/monounsaturated phospholipids (S/M-PL), measured in control A375 cells (Ctrl) and cells treated for 24 h with eupatilin (EUP) and xanthomicrol (XAN) 10 and 25 μM. The chromatographic region of each lipid class was assigned using a mixture of standard saturated/monounsaturated (PC: 16:0/16:0, 18:1/18:1, 16:0/18:1, 18:1/16:0) and polyunsaturated phosphatidylcholines (PC: 16:0/18:2, 16:0/20:4, 18:2/18:2; 20:5/20:5). (**B**) Values (expressed as % controls) of PL and FC measured in A375 control cells and cells treated with EUP and XAN (10 and 25 μM). All data are presented as mean (*n* = 6) and standard deviation. For each data series: *** = *p* < 0.001, ** = *p* < 0.01, * = *p* < 0.05 versus Ctrl; °°° = *p* < 0.001, °° = *p* < 0.01 for EUP and XAN 25 μM versus the respective 10 μM-treated cells (One-way ANOVA and Bonferroni post Test).

Figure 6A shows the reversed-phase HPLC-DAD/ELSD chromatographic profile of lipid compounds (P-PL, S/M-PL, and FC) obtained for A375 control cells and cells 24 h-treated with EUP and XAN 10 and 25 μM.

The polar lipid profile of control A375 melanoma cells was characterized by a peak of FC and two main peaks of PL, corresponding to saturated/monounsaturated PL (S/M-PL) and polyunsaturated PL (P-PL). Figure 6B shows the PL and FC values (as % controls) determined in control A375 cells and melanoma cells 24 h-incubated with EUP and XAN (10 and 25 μM). The treatment with both flavones at 10 μM induced a significant decrease in the peak areas of S/M-PL in comparison to untreated cells and a correlated rise in FC amount, while a moderate increase in the % of P-PL (*p* < 0.05) was observed only in XAN-treated cells. The decrease in the % amount of S/M-PL and the increase in % of FC were more marked (*p* < 0.001 versus untreated cells and for each compound versus the respective 10 μM-treated cells) at the dose of 25 μM. Both compounds significantly affected the lipid profile in cancer A375 cells, with a similar modulatory effect.

**Figure 7:**
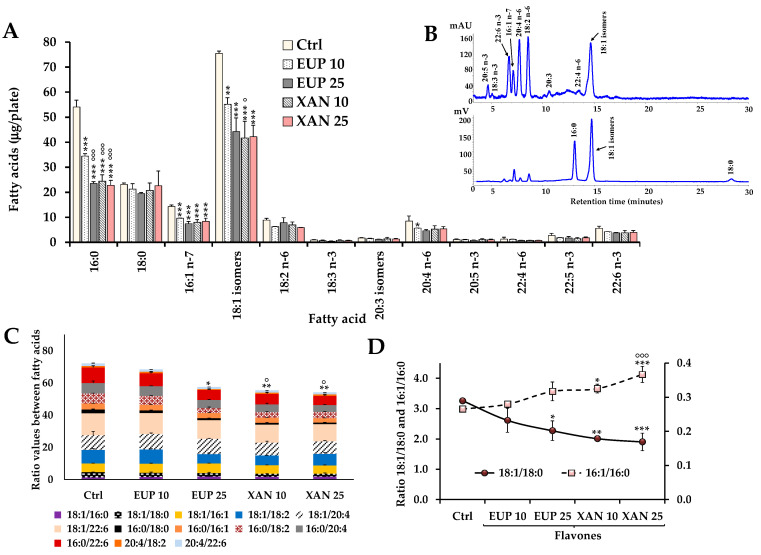
Values (expressed as μg/plate) of the main saturated and unsaturated fatty acids measured in control A375 melanoma cells (Ctrl) and cells treated for 24 h with eupatilin (EUP) and xanthomicrol (XAN) 10 and 25 μM (**A**) and the chromatographic profile of the control A375 cells measured by DAD (at 200 nm) and ELSD detection (**B**). Values of the ratios among the main FA measured in A375 control cells and cells treated with EUP and XAN, with each stacked bar chart representing the sum of FA ratios determined in each cell sample (**C**). Values of 16:1 n-7/16:0 and 18:1 n-9/18:0 ratios measured in control and treated A375 cells (**D**). All data are presented as mean (*n* = 6) and standard deviation. *** = *p* < 0.001, ** = *p* < 0.01, * = *p* < 0.05 versus Ctrl; °°° = *p* < 0.001, ° = *p* < 0.05 versus cells treated with EUP 10 μM. Statistical significance of differences was assessed by One-way ANOVA and Bonferroni post Test.

Then, cell total lipid extracts were subjected to mild saponification and the saponifiable fraction of cell samples was used for the determination of the cell total FA profile (TFA). Values of the main saturated (SFA) and unsaturated FA (UFA), expressed as μg/plate, measured in control melanoma cells and A375 cells incubated for 24 h with EUP and XAN (10 and 25 μM) are reported in Figure 7A.

The FA chromatographic profile of control A375 cancer cells, measured with DAD (at 200 nm) and ELSD detection, is reported in Figure 7B. A375 control cells were characterized by a high quantity of 18:1 isomers (75.6 ± 0.9 μg/plate, 38.2% of TFA; mainly 18:1 n-9), palmitic acid 16:0 (54.6 ± 2.7 μg/plate, 27%), stearic acid 18:0 (23.2 ± 0.5 μg/plate, 11.7%), and palmitoleic acid 16:1 n-7 (14.3 ± 0.6 μg/plate, 7.2%). The most abundant polyunsaturated FA (PUFA) were linoleic acid 18:2 n-9 (8.9 ± 0.7 μg/plate, 4.5%) and arachidonic acid 20:4 n-6 (8.5 ± 2.0 μg/plate, 4.3%). The treatment of cancer A375 cells with EUP and XAN for 24 h (Figure 7A) greatly affected the FA profile in comparison to control cells. EUP induced a significant reduction in the % cell amount of 18:1 n-9, 16:0, and 16:1 n-7 versus control cells at both doses, although its effect was more marked at 25 μM. XAN was more active than EUP in decreasing the cellular level of 18:1 n-9, 16:0, and 16:1 n-7 at 10 μM, however, the two flavones showed similar modulatory effects on cancer cell FA levels at 25 μM. No significant changes were observed in the % levels of other FA in EUP- and XAN-treated cells versus untreated cells.

The cell treatment with DMSO, the vehicle for the solubilization of flavones, did not affect cell FA profile versus untreated cells.

The FA 18:1 n-9, 16:0, 18:0, 16:1 n-7, 18:2 n-6, 20:4 n-6, and 22:6 n-3 emerged as the most abundant FA in A375 cancer cells. Therefore, we decided to calculate the ratios between these main FA to overcome eventual differences in the FA content among cell samples due to differences in the absolute number of cells. Recent studies have proposed FA ratios (dimensionless quantities) as a suitable way to effectively replace the original FA data set [34]. Figure 7C shows the total values of the ratios between the main FA measured in control A375 cells and cells treated for 24 h with EUP and XAN (10 and 25 μM).

Control A375 cells showed a specific profile of FA ratios. High ratio values were measured for 18:1 n-9/22:6 n-3, 16:0/22:6 n-3, 18:1 n-9/20:4 n-6, 18:1 n-9/18:2 n-6, 16:0/20:4 n-6, and 16:0/18:2 n-6. A significant decrease in the total values of FA ratios (as the sum of all FA ratios determined in each cell sample) was observed in cells treated with EUP 25 μM (*p* < 0.05) and XAN 10 and 25 μM (*p* < 0.01) in comparison to untreated cells, confirming the modulatory effect of these flavones on FA metabolism.

The value of the ratio 18:1 n-9/18:0 greatly decreased (Figure 7D) in XAN-treated cells versus control cells (characterized by the value of 3.3 ± 0.1). Melanoma cells treated with XAN 10 and 25 μM showed 18:1 n-9/18:0 ratio values of 2.0 ± 0.1 (*p* < 0.01 versus control cells) and 1.9 ± 0.3 (*p* < 0.001), respectively. A significant rise in the 16:1 n-7/16:0 ratio value was also evidenced in cells treated with XAN 10 μM (*p* < 0.05 versus controls) and 25 μM (*p* < 0.001). A similar effect on 16:1 n-7/16:0 ratios and 18:1 n-9/18:0, even though less marked, was evidenced in 25 μM EUP-treated cells.

### 3.4. Effect on Mitochondrial Membrane Potential and Apoptosis

Finally, the effect of EUP and XAN on mitochondrial membrane potential and apoptosis was evaluated in A375 melanoma cells.

**Figure 8:**
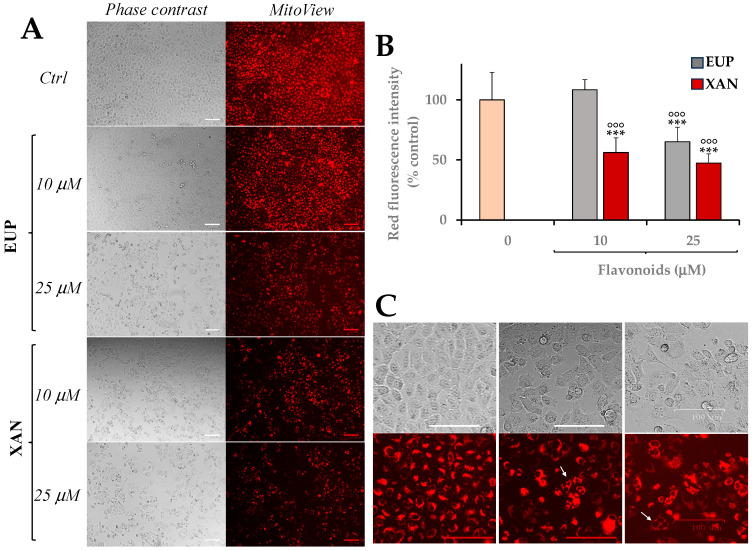
Phase contrast and red emission images as revealed by MitoView 633 fluorescence of control A375 cells (Ctrl) and melanoma cells 24 h-treated with eupatilin (EUP) and xanthomicrol (XAN) 10 and 25 μM (bar = 100 μm) (**A**). Mitochondrial membrane potential variations (intensity of red emission fluorescence expressed as % controls) after EUP and XAN treatment as resulted after image analysis (**B**). White arrows display multinucleated figures in red emission images measured in cells after 24 h-treatment with 25 μM EUP and XAN (**C**). All data are presented as mean (*n* = 6) and standard deviation. *** = *p* < 0.001 versus Ctrl; °°° = *p* < 0.001 versus cells treated with EUP 10 μM. Statistical significance of differences was assessed by One-way ANOVA and Bonferroni post Test.

Figure 8A shows images of phase contrast and red emission (as revealed by MitoView 633 fluorescence) of A375 control cells and melanoma cells 24 h-incubated with EUP and XAN at the concentrations of 10 and 25 μM. Quantitative data of red fluorescence intensity (expressed as a percentage of control cells) measured in control cells and flavones-treated cells, after image analysis, are depicted in Figure 8B.

The 24 h-treatment with XAN induced, at both concentrations, a significant (*p* < 0.001) marked decrease (44% and 53% reduction at 10 and 25 μM, respectively) in the red fluorescence signal versus control cells, indicating a depolarization of mitochondrial membrane potential. Cells treated with EUP 10 μM showed a red fluorescence emission similar to control cells, while the dose of EUP 25 μM induced a noticeable mitochondrial depolarization, exhibiting a significant 35% reduction in the red fluorescence signal in comparison to control cells (*p* < 0.001).

Phase contrast and MitoView 633 fluorescence images (Figure 8C) show that control A375 cells were small, packed, and mononucleated. The treatment with both EUP and XAN doses elicited a remarkable decrease in cell density and the occurrence of a high number of rounded cells (apoptotic cells). Moreover, non-rounded cells characterized by multiple nuclei were frequently observed at the highest EUP and XAN concentration, discernible in MitoView 633-stained cells.

Finally, the effect of the two flavones on apoptosis induction was assessed by staining A375 melanoma cells with NucView 488, a substrate of the enzyme caspase-3, able to detect the activity of caspase-3/7 inside cells [45].

**Figure 9:**
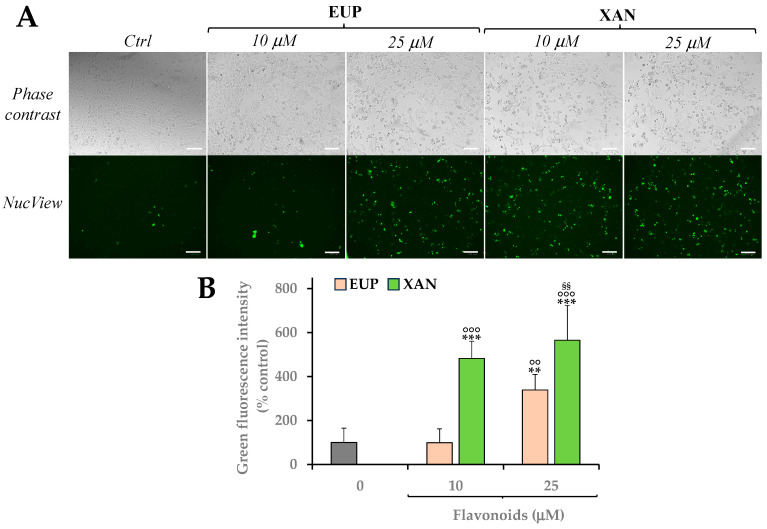
Phase contrast and green emission images (bar = 100 μm) as revealed by NucView 488 fluorescence of control A375 cells (Ctrl) and melanoma cells 24 h-treated with eupatilin (EUP) and xanthomicrol (XAN) 10 and 25 μM (**A**). Apoptosis induction (intensity of green emission fluorescence expressed as % controls) after EUP and XAN treatment as resulted after image analysis (**B**). All data are presented as mean (*n* = 6) and standard deviation. *** = *p* < 0.001, ** = *p* < 0.01 versus Ctrl; °°° = *p* < 0.001, °° = *p* < 0.01 versus cells treated with EUP 10 μM; ^§§^ = *p* < 0.01 versus cells treated with EUP 25 μM. Statistical significance of differences was assessed by One-way ANOVA and Bonferroni post Test.

Figure 9 shows images of phase contrast and green emission (as revealed by NucView 488 fluorescence) (Figure 9A) and quantitative data of green fluorescence intensity (expressed as a percentage of control cells) measured in A375 control cells and cells 24 h-incubated with EUP and XAN at the doses of 10 and 25 μM (Figure 9B).

Cells 24 h-treated with EUP 10 μM showed a green fluorescence emission similar to control cells, while the cell incubation with EUP 25 μM induced a significant (*p* < 0.01) rise in the number of rounded and green-fluorescent apoptotic cells in comparison to control cells (value of 339% of controls).

The 24 h-treatment with XAN induced, at both concentrations, a significant (*p* < 0.001) marked increase, versus control cells, in the number of NucView 488-stained cells, with values of 482% and 566% of green fluorescence intensity at 10 and 25 μM, respectively.

## 4. Discussion

Various natural isolated metabolites and extracts from plants have revealed therapeutic effects on different types of cancers [2,50,51]. Despite the anticancer properties of phytochemicals, medication resistance, systemic toxicity, and limited absorption represent significant problems in clinical trials [50]. Moreover, the detailed mechanisms underlying the anticancer properties of phytochemicals need to be further explored to facilitate the development of natural product-based anticancer agents [51].

Methoxylated flavones have demonstrated chemopreventive properties superior to unmethylated flavonoids due to their lower polarity, which enhances transport through biological membranes and oral bioavailability [9,10,11]. The antitumor activity of polymethoxylated flavones has been associated with their ability to modulate various molecular targets and signaling pathways in cancer cells, determining cytotoxic and antiproliferative effects, apoptosis, and cell cycle arrest [9,10,11,12].

The natural methoxylated flavones EUP and XAN are chemical analogs, characterized by certain lipophilicity as indicated by their computed chemical and physical properties such as the number of H-bonds formed, the log P3 value, and the topological polar surface area (TPSA) (Table 1) [15]. We previously demonstrated the ability of EUP and XAN to target lipid metabolism in cancer HeLa cells [13,14].

In this manuscript, the modulatory effect of EUP and XAN on lipid profile was investigated and compared for the first time in cutaneous melanoma A375 cells, together with the ability of the two methoxylated flavones to affect cell growth, cell morphology, apoptosis, mitochondria membrane potential, and intracellular ROS generation.

Initially, EUP and XAN cytotoxicity was assessed in A375 cancer cells after 24 h of incubation. Both compounds significantly affected viability in A375 cells, with XAN showing higher potency than EUP at the lowest tested concentrations, but similar cytotoxicity from the dose of 25 μM. Previous studies evidenced the superior cytotoxicity of XAN in comparison to EUP in human cervical cancer cells HeLa after 24 h of incubation, and IC_50_ values of 182 μM and >200 μM were reported for XAN and EUP, respectively [13,14]. An IC_50_ value of 35 μg/mL (101.7 μM) was previously reported for XAN in 4T1 cancer cells after 24 h-incubation [30], whereas IC_50_ values ranging from 4.5 to 40.6 μg/mL (approximately 13–124 μM) were determined in several malignant cells (AGS, WEHI-164, HL60, SaOs-2, and HT29) after 72 h of incubation [28] (Table 2).

According to our results, a previous study evidenced the cytotoxicity of EUP in A375 cancer cells after 24 h of incubation, with a cell growth inhibitory effect of approximately 50% at 200 μM [52]. The cytotoxicity value obtained for EUP in our experimental conditions was lower than those values previously observed in human endometrial cancer Hec1A [22], KLE [22], and HeLa [23] cells after 48 h-incubation with this methoxylated flavone (Table 2). The well-known anticancer flavone QRC [13,49] showed in A375 cells a cytotoxicity pattern similar to EUP and XAN in the range of 25–200 μM after 24 h of incubation.

In our experimental conditions, both compounds were not able to induce a 50% reduction in cell death at the concentrations tested, due to the short time of incubation (24 h), the high drug resistance of A375 melanoma cells [38], and the limits of cell tolerability for the vehicle used to dissolve the lipophilic flavones.

Marked changes in the cell morphology of A375 cancer cells 24 h-treated with both flavones were evidenced by microscopic observation, such as a reduced cell density, an increase in the number of rounded and granulated cells, and membrane blebbing, highlighting evident signs of an apoptotic process.

EUP and XAN cytotoxicity was then assessed in HaCaT cells, a normal human skin keratinocyte cell line, after 24 h of incubation. XAN exhibited in keratinocytes higher toxicity than EUP, as observed in A375 cells, and it was less toxic in HaCaT cells than in A375 cells only at the lowest tested dose (2.5 μM). Certain cytotoxicity was previously observed for XAN in normal human fetal foreskin fibroblasts HFFF-P16 [28] and 3T3 fibroblasts [14]. EUP showed lower cytotoxicity versus normal HaCaT skin keratinocytes than A375 cells in the range 2.5–25 μM, evidencing a certain selectivity towards skin malignant cells at low doses, as previously observed in 3T3 fibroblasts [14]. The absence of an elevated selective viability reduction in EUP and XAN in A375 cells compared to normal keratinocytes was probably attributable to the high drug resistance of melanoma cells [38]. A previous study evidenced the superior cytotoxic effect of the anticancer flavonoid luteolin (3,4,5,7-tetrahydroxy flavone) in HaCaT cells (IC_50_ value of 37.17 μM) than in A375 cells (IC_50_ value of 115.1 μM) after 24 h-incubation [53].

The prooxidant effect of several flavonoids on the cellular level has been demonstrated to partially contribute to their anticancer activity [54,55]. The cytotoxic effect of several flavonoids against cancers has been primarily ascribed to their ability to evoke excessive oxidative stress [53,55]. A marked time-dependent increase in the intracellular ROS level was observed in A375 melanoma cells during 3 h-treatment with EUP and XAN versus control cells, and the prooxidant effect was more marked at 10 μM for both compounds. It is worth noting that the two methoxylated flavones did not exert a prooxidant effect in normal skin keratinocytes during 3 h of incubation, evidencing a significant protective effect versus control cells at 25 μM, ascribable to their antioxidant properties [9,13,18,20,21].

Our data evidenced ROS generation as another potential toxicity mechanism of EUP and XAN in melanoma cells. Previous work reported that the EUP anticancer activity is partly mediated through its prooxidant activity [18].

The prooxidant effects in cancer cells of several radical-producing agents, used in antitumor treatments, are related to their interference with mitochondrial functions [48]. Flavonoids can activate apoptotic, autophagic, and necroptotic pathways in cancer cells by the induction of excessive ROS accumulation [54,55]. A study conducted on four typical flavonoids (apigenin, quercetin, chrysin, and diosmetin) demonstrated that high-dose flavonoids markedly triggered cell death via oxidative stress (as evidenced by upregulated ROS and MDA and downregulated SOD activity) and apoptosis, cell cycle arrest, accumulated mitochondrial superoxide, impaired mitochondrial function, and decreased ATP synthesis emerged as the underlying mechanism of cell death [54]. An increase in ROS by giving oxidant treatments or by removing cellular antioxidant systems has been proposed as a therapeutic approach to trigger cell death in melanoma genesis/progression [41].

Then, the modulatory effect of EUP and XAN on lipid profile in cutaneous melanoma cells was investigated and compared for the first time in cancer A375 cells. The incubation of A375 melanoma cells with both flavones triggered noticeable alterations in the phospholipid/cholesterol ratio, with a significant decrease in the % amount of S/M-PL. In our experimental conditions, the most abundant FA measured in control melanoma A375 cells were 18:1 n-9 and 16:0, followed by a lower amount of 18:0 and 16:1 n-7. The 24 h-incubation of cancer A375 cells with EUP and XAN induced a significant reduction in the cell level of 18:1 n-9, 16:0, 16:1 n-7, and in the total values of FA ratios in comparison to control cells. The reduced level of S/M-PL observed in treated cells was mainly ascribable to a decrease in the level of PL containing 16:0 and 18:1 n-9. A marked decrease in the 18:1 n-9/18:0 ratio value and a significant increase in the 16:1 n-7/16:0 ratio value were evidenced in A375 cells treated with XAN and EUP versus control cells.

Our data confirmed results previously obtained for XAN and EUP in HeLa cancer cells [13,14]. The two flavones exhibited the ability to modulate HeLa cell FA and PL profiles, with a remarkable decrease in the levels of 16:0 and 18:1 n-9, and a reduction in the S/M-PL amount linked to an increase in the FC percentage value [13,14].

High rates of exogenous FA uptake and de novo lipid synthesis have been reported in cancer cells and several studies have revealed that most of the lipogenic enzymes are upregulated/activated in tumor cells [31,32,34,35,36]. Lipid metabolism is considered a promising anti-metastatic drug target in cancer therapies [31,32,34,35,36]. Several antitumor drugs act through the membranes by inhibiting the expression of lipogenic enzymes involved in FA metabolism such as stearoyl-CoA desaturase (SCD), FA synthase (FAS), and ATP-citrate lyase [31,32,34,35,36]. FAS is a multifunctional polypeptide enzyme that catalyzes de novo synthesis of 16:0 [35,36]. The inhibition of the FAS expression can decrease the proliferation/growth of cancer cells, resulting in tumor apoptosis; therefore, FAS is considered a promising target for drug discovery [32,35,36]. Anticancer flavones like luteolin, quercetin, and amentoflavones are inhibitors of the enzyme FAS [36]. The stearoyl-CoA desaturase (SCD), an integral membrane protein, catalyzes the rate-limiting step in the formation of 18:1 n-9 and/or 16:1 n-7 from stearoyl-(18:0) or palmitoyl-CoA (16:0) [32,35]. SCD is significantly increased in tumors and SCD-mediated desaturation of FA may represent an important step for cancer cell survival [32]. The major products of SCD, oleic and palmitoleic acids, are key substrates for the generation of phospholipids, triglycerides, and cholesterol esters [34,35]. Cancer cells depend on lipid synthesis pathways for growth/survival because their high proliferation requires large amounts of lipids as metabolic fuels through oxidation in mitochondria and building blocks for biological membranes [31,34,56,57]. The generation of lipid membranes and membrane fluidity maintenance, essential to cell proliferation, often require de novo synthesis of MUFA in cancer cell lines [34,35,57]. Treatment with SCD inhibitors was found to disrupt the balance between MUFA and SFA and reduce the survival rate of tumor cells [32]. Flavonoids like the tetrahydroxyflavone kuwanon C showed potential inhibition of SCD [58].

XAN and EUP significantly affected lipid metabolism in A375 melanoma cells, as indicated by the changes observed in the PL profile and FA amount/ratios. It has been reported that several lipid pathways, such as the de novo synthesis, elongation, and desaturation of FA, and the synthesis of glycerophospholipids are altered in melanoma cells [42]. The most prominent phenomenon in melanoma cells is an increased rate of lipogenesis [42]. The 16:0 decrease induced by EUP and XAN in cancer A375 cells was compatible with a possible FAS inhibition [13,14]. Moreover, the reduction in MUFA (18:1 n-9 and 16:1 n-7) and the marked decrease in the 18:1 n-9/18:0 ratio value observed in melanoma cells treated with both flavones could be due to an inhibition of SCD. The increase observed in the value of the 16:1 n-7/16:0 ratio is probably derived from a more marked 16:0 decrease. The 18:1 n-9 and 16:0 depletion greatly influenced the phospholipid composition in A375-treated cells, leading to a reduction in the S/M-PL amount together with a rise in the FC % level, and a decrease in the SFA/PUFA and MUFA/PUFA ratios.

The 24 h-incubation of cancer melanoma A375 cells with the flavones EUP and XAN induced a marked modulation of the cell lipid profile probably through the inhibition of lipogenesis and FA desaturation, and the alteration of the PL composition. The biophysical/functional properties of cancer cell membranes are severely affected by changes in lipid components that induce variations in membrane structure, organization, and fluidity, consequently perturbing membrane lipid rafts and protein dynamics [13,14,34,35]. Moreover, abrogation of lipid synthesis through inhibition of lipogenic enzymes seriously impacts the cancer cell metabolism, inducing a decrease in cell growth/proliferation and an increased apoptosis [13,14,56,57]. Therefore, variations in lipid components induced by EUP and XAN in A375 cancer cells could cause changes in the organization, structure, and fluidity of lipid membranes, modulating the membrane interaction with pivotal signaling proteins [13,14]. Modification of signaling pathways regulated by these proteins resulted in a melanoma cell viability decrease and the induction of apoptosis as indicated by the MTT assay and morphological observation.

Finally, the effect of the two methoxy flavones on mitochondria membrane potential and apoptosis was assessed in melanoma A375 cells after 24 h of incubation. The two methoxylated flavones induced a decrease in the mitochondrial membrane potential monitored by MitoView 633, a far-red fluorescent mitochondrial dye. Phase contrast and red fluorescence images allowed us to detect a noteworthy cell density decrease and the occurrence of rounded cells (apoptotic cells). Moreover, MitoView 633-staining showed the existence of non-rounded cells characterized by multiple nuclei, discernible in A375 cells pre-incubated with both EUP and XAN. Previous studies reported the ability of EUP [13,18,25] and XAN [14] to induce mitochondrial membrane depolarization in several cancer cell lines. The presence of A375 cells with multiple nuclei is an index of abnormal mitosis, a delayed mitosis-linked cell death, involved in the effects of several anticancer extracts/compounds [13,59,60]. Mitotic catastrophe is due to abnormal mitotic events that determine spontaneous premature chromosome condensation and cell division with the characteristic features of polynucleated cells, presenting two or more nuclei residues from a deficient separation during cytokinesis [13,59,60].

Changes in the mitochondrial inner membrane potential occur during apoptosis [47], and the expression of caspase-3 protein promotes the acceleration of the mitochondrial apoptotic pathway [45]. Therefore, the effect of EUP and XAN on apoptosis induction was assessed by staining A375 melanoma cells with NucView 488, a substrate of the enzyme caspase-3, able to detect caspase-3/7 activity inside cells [45]. Previous studies demonstrated the capacity of EUP [13,18,24] and XAN [9,14,27,30] to induce apoptosis in various cancer cell lines. The 24 h-treatment of melanoma A375 cells with XAN induced a noticeable increase in the number of green-fluorescent cells in comparison to control cells at both tested concentrations, whereas EUP induced a significant increase in the number of rounded/green-fluorescent apoptotic cells at 25 μM. In previous research conducted in cancer HeLa cells, we demonstrated, by flow cytometry, immunofluorescence, and fluorescence microscopy, the EUP capacity (in the dose range 10–50 μM, 24 h of incubation) to induce apoptosis (presence of rounded cells, apoptotic bodies, and membrane blebbing) and abnormal mitosis with multinucleation (mitotic catastrophe) [13]. Moreover, we recently assessed the occurrence of apoptosis, by microscopic observation and analysis of the cell cycle, in cancer HeLa cells after 24 h of incubation with XAN at the dosage range of 5–50 μM [14]. However, to the best of our knowledge, this study highlighted for the first time the XAN-induction of abnormal mitotic events in cancer cells.

Taken together, the results of this study demonstrated the ability of EUP and XAN to induce cytotoxicity, morphological alteration, lipid profile modulation, ROS generation, apoptosis, mitochondrial membrane depolarization, and mitotic catastrophe in melanoma A375 cells. The two flavones exhibited similar biological profiles; however, certain dose-dependent differences were observed, as previously measured in cancer HeLa cells [14]. EUP and XAN are chemical analogs, with identical values of lipophilicity (log P3 = 2.9), TPSA, and total number of H-bonds [15]. These properties greatly influence the interaction between the flavonoids-lipid bilayer and flavonoids-enzymes [61]. The lipophilicity of XAN and EUP could permit the interaction of these flavones with the cell membrane, the induction of changes in membrane fluidity/organization, and the alteration of membrane-mediated cell signaling pathways involved in cell lipid metabolism, growth/proliferation, and apoptosis in A375 melanoma cells. Different mechanisms could contribute to the XAN and EUP antitumor properties in A375 cancer cells; however, the exact sequence of events in their anti-tumor efficacy was very difficult to determine.

## 5. Conclusions

Melanoma is one of the most therapy-resistant types of cancer; therefore, the development of new therapeutic approaches is strongly mandatory. Our data demonstrated that the incubation of melanoma A375 cells for 24 h with EUP and XAN induced a marked modulation of the cell lipid profile, possibly through the reduction in lipogenesis and FA desaturation, which led to an altered PL biosynthesis and FA profile. Moreover, the two flavones exerted anticancer effects in A375 human malignant melanoma cells through the induction of cytotoxicity, apoptosis/mitotic catastrophe, ROS generation, mitochondrial depolarization, and morphological alteration.

In conclusion, the present study highlighted the potential role of EUP and XAN as novel therapeutic strategies in melanoma; however, further studies are needed to assess their anticancer effect in in vitro tumor models and their safety profile in different types of normal cells.

## Figures and Tables

**Table 1:** Computed chemical and physical properties of eupatilin, xanthomicrol, and quercetin [15].

Flavone	Log P3	Number of H-Bonds ^1^	Topological Polar Surface Area ^2^
Eupatilin	2.9	9	94.4
Xanthomicrol	2.9	9	94.4
Quercetin	1.5	12	127.0

^1^ Log P3: partition coefficient for an octanol–water mixture, computed by XLogP3 3.0. ^2^ Computed by Cactvs 3.4.8.18.

**Table 2:** Cytotoxic activity of EUP and XAN in several normal and cancer cells from literature data.

Compound	Cell Line	Method	Incubation Time (h)	IC_50_^a^	Viability % (at 10 μM)	Literature Reference
EUP	Cancer HeLa	MTT	24	>200 μM	89	[13]
	Cancer HeLa	CCK-8 kit	48	-	70	[23]
	Cancer Ect1/E6E7	CCK-8 kit	48	-	90	[23]
	Cancer Hec1A	MTT	48	82.2 μM	-	[22]
	Cancer KLE	MTT	48	85.5 μM	-	[22]
	Normal HES	MTT	48	61.2 μM	-	[22]
	Normal HESC	MTT	48	65.5 μM	-	[22]
	Normal 3T3	MTT	24	>200 μM	90	[14]
XAN	Cancer HeLa	MTT	24	182 μM	72	[14]
	Cancer AGS	MTT	72	4.5 μg/mL	-	[28]
	Cancer HT29	MTT	72	42.6 μg/mL	-	[28]
	Cancer HL60	MTT	72	38.5 μg/mL	-	[28]
	Cancer SaOs-2	MTT	72	40.6 μg/mL	-	[28]
	Cancer WEH-I164	MTT	72	32.8 μg/mL	-	[28]
	Cancer 4T1	MTT	24	35.0 μg/mL	-	[30]
	Normal HFFF-P16	MTT	72	55.9 μg/mL	-	[28]
	Normal 3T3	MTT	24	>200 μM	92	[14]

^a^ IC_50_ value: the concentration of compound that induces a 50% cell viability decrease.

## Data Availability

Data of the current study are available from the corresponding author upon reasonable request.
